# Efficient Olfactory Coding in the Pheromone Receptor Neuron of a Moth

**DOI:** 10.1371/journal.pcbi.1000053

**Published:** 2008-04-25

**Authors:** Lubomir Kostal, Petr Lansky, Jean-Pierre Rospars

**Affiliations:** 1 Institute of Physiology, Academy of Sciences, Prague, Czech Republic; 2 INRA, UMR 1272 Physiologie de l'Insecte, Versailles, France; University College London, United Kingdom

## Abstract

The concept of coding efficiency holds that sensory neurons are adapted, through both evolutionary and developmental processes, to the statistical characteristics of their natural stimulus. Encouraged by the successful invocation of this principle to predict how neurons encode natural auditory and visual stimuli, we attempted its application to olfactory neurons. The pheromone receptor neuron of the male moth *Antheraea polyphemus*, for which quantitative properties of both the natural stimulus and the reception processes are available, was selected. We predicted several characteristics that the pheromone plume should possess under the hypothesis that the receptors perform optimally, i.e., transfer as much information on the stimulus per unit time as possible. Our results demonstrate that the statistical characteristics of the predicted stimulus, e.g., the probability distribution function of the stimulus concentration, the spectral density function of the stimulation course, and the intermittency, are in good agreement with those measured experimentally in the field. These results should stimulate further quantitative studies on the evolutionary adaptation of olfactory nervous systems to odorant plumes and on the plume characteristics that are most informative for the ‘sniffer’. Both aspects are relevant to the design of olfactory sensors for odour-tracking robots.

## Author Summary

Efficient coding is an overarching principle, well tested in visual and auditory neurobiology, which states that sensory neurons are adapted to the statistical characteristics of their natural stimulus - in brief, neurons best process those stimuli that occur most frequently. To assess its validity in olfaction, we examine the pheromone communication of moths, in which males locate their female mates by the pheromone they release. We determine the characteristics of the pheromone plume which are best detected by the male reception system. We show that they are in agreement with plume measurements in the field, so providing quantitative evidence that this system also obeys the efficient coding principle. Exploring the quantitative relationship between the properties of biological sensory systems and their natural environment should lead not only to a better understanding of neural functions and evolutionary processes, but also to improvements in the design of artificial sensory systems.

## Introduction

According to the ‘efficient-coding hypothesis’ [1], the sensory neurons are adapted to the statistical properties of the signals to which they are exposed. Because not all signals are equally likely, sensory systems should best encode those signals that occur most frequently. This idea was first tested by Laughlin [2] in a pioneering study of first order interneurons in the insect compound eye, the large monopolar cells, which code for contrast fluctuations. He showed that the response function of these graded potential cells, measured by intracellular recording, approximates the cumulative probability distribution function of contrast levels measured in the natural fly's habitat with a photodiode.

The efficient coding hypothesis has been much studied in the visual system [2]–[7]; reviewed in [8] and to a lesser extent in the auditory system [9],[10]. However, it has been rarely discussed in the context of olfactory sensory neurons [11],[12].

**Figure 1:**
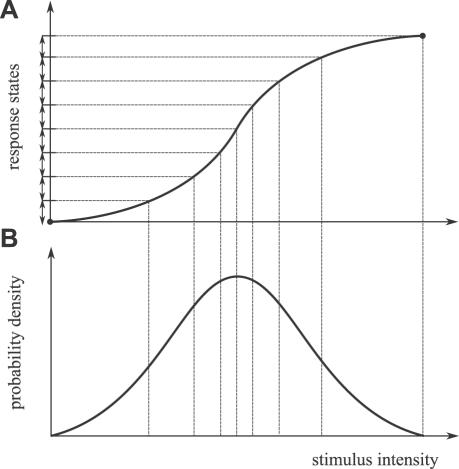
Amount of information transferred by a neuron in the case where all response states are equiprobable. (A) Stimulus-response function. The amount of transferred information is limited by the finite range of possible response states. Due to the non-linearity of the stimulus-response function, each response state encodes different relative changes in stimulus intensity. (B) Corresponding probability density function (pdf). Maximum information is transferred if all response states are used equally, i.e., if the area under the stimulus pdf is equal for each response state, as shown. In the limit of vanishingly small response states, the optimal stimulus CDF corresponds to the (normalized) stimulus-response function (adapted from [2]).

With a nonlinear stimulus-response function, the neuron encodes differently an equal change in stimulus intensity depending on the actual concentration (Figure 1A). The key question is, how should a neuron weigh its input so as to transfer as much information as possible? Information theory [13],[14] provides the solution. In the simplest scenario (with no other constraints on the response range), the inputs should be encoded so that all responses are used with the same frequency [2]. The optimal stimulus statistics is given by the stimulus probability distribution (Figure 1B), which is obtained directly from the stimulus-response curve. This simple solution, however, does not hold in the case of olfaction because of the large differences in reaction time at different stimulus concentrations. This is a major difference with respect to Laughlin's approach, in which all response states were assumed to be equiprobable.

In this paper, we paralleled Laughlin's approach [2], adapting his method to suit the specificity of olfaction. We chose a well studied olfactory receptor neuron, the pheromone receptor neuron of male moths, to investigate its adaptation to the natural signal it processes, the sexual pheromone emitted by conspecific females. To our knowledge this neuron and its stimulus provide the only example in olfaction for which enough data are available on the odorant plume and the neuron transduction mechanisms to make a quantitative comparison possible between the predicted optimum signal and the natural signal.

**Figure 2:**
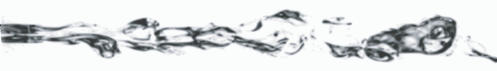
Visualization of a pheromone plume. The figure is extracted and adapted from a digitized image of a smoke plume filmed in a wind tunnel 1 m across and 2 m long with source on the left side [43]. Though the average pheromone concentration in the air decreases with distance, high pheromone concentrations can be found relatively far from the source due to the imperfect mixing of odorant with air. The signal detected by both moving and stationary detectors is therefore always intermittent, consisting of pulses of relatively undiluted pheromone.

Flying male moths rely on the detection of pheromone molecules released by immobile conspecific females for mating. The atmospheric turbulence causes strong mixing of the air and creates a wide spectrum of spatio-temporal variations in the pheromonal signal (Figure 2). The largest eddies are hundreds of metres in size and may take minutes to pass a fixed point, while the smallest spatial variations are less than a millimetre in size and last for milliseconds only [15],[16]. Due to inhomogeneous mixing, a very high concentration of pheromone can be found in a wide range of distances from the source, though their frequency decreases with distance [15]. Because of its complicated and inhomogeneous structure, the description of the plume must rely on statistical methods, notably the histogram of the fluctuations in pheromone concentration [15]–[19]. These fluctuations are essential for the insect to locate the source of the stimulus. Experiments in wind tunnels showed that moths would not fly upwind in a uniform cloud of pheromone [20]–[22]. Characteristics like the frequency and intensity of the intermittent stimulation play a key role in maintaining the proper direction of flight [23].

The goal of this paper is to present arguments specifying in which sense the perireception and reception processes occuring in pheromone olfactory receptor neurons (ORNs) can be considered as optimally adapted to their natural stimulus. Although, in the light of previous studies on similar sensory neurons, the ORN may be considered *a priori* as adapted to the pheromone plume, the exact nature of this adaptation and its proof are more challenging questions. Despite widespread agreement that environmental statistics must influence neural processing [24], precise quantification of the link proved difficult to obtain [8]. So, the main aim of this paper was to identify the specific characteristics to which the pheromone ORN is adapted and to provide quantitative evidence for their adaptation. We proceeded in two steps. First, using the statistical theory of information, we predicted the characteristics of the optimal pheromonal signal that the ORN is best capable of encoding based on the properties of the initial steps of signal transduction. Second, we compared these theoretically-derived properties with statistical characteristics most often determined in experimental measurements, i.e., the probability distribution function of the fluctuations in pheromone concentration, the spectral density function of the stimulation course and the intermittency of the odorant signal.

## Results

### Model of Pheromone Reception

Pheromone components are detected by specialized ORNs located in the male antenna. We considered a specific ORN type of the moth *Antheraea polyphemus* detecting (E,Z)-6,11-hexadecadienyl acetate, the major component of the sexual pheromone in this species, for which a wealth of precise information is available (reviewed in [25]). The pheromone molecules are adsorbed on the cuticle, diffuse inside the sensory hair to the neuron membrane and are thought to be enzymatically deactivated [25] then degraded. The initial cell response is triggered by the binding of the pheromone molecules to the receptor molecules borne by the dendritic membrane and the ensuing receptor activation. A cascade of events follows, amplifying this initial response and finally leading to the generation of a train of action potentials conveyed to the brain. The pheromone concentration at each instant determines the ORN response. However the extreme temporal variability of pheromone concentration in plumes prevents a full description of stimulus-response relationships by direct electrophysiological measurements. For this reason we based our study on a model of perireception and reception processes describing how any stimulus (concentration of pheromone in the air) is transformed into the receptor response (concentration of activated receptors). This model, based on extensive biochemical, radiochemical and electrophysiological experiments, was developed by Kaissling and coworkers [25],[26]. It involves the following system of chemical reactions:
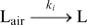
(1)

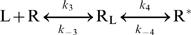
(2)

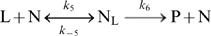
(3)The network includes (1) the translocation of the ligand from the air (input pheromone signal L_air_) to the hair lumen (L); (2) the reversible binding of L to receptor R and the reversible change of the complex R_L_ to an activated state R^*^ (output signal); (3) the reversible binding of L to a deactivating enzyme N and its deactivation to product P which is no longer able to interact with the receptor.

The concentrations of individual components in the network 1–3 are denoted by square brackets and the concentration values are functions of time. For simplicity we omit here the explicit dependence on the time variable *t* and adopt the following notation for the individual concentrations: *L*_air_ = [L_air_](*t*),  = [L](*t*), *R* = [R](*t*), *R*_L_ = [R_L_](*t*), *R* ^*^ = [R^*^](*t*), *N* = [N](*t*), *P* = [P](*t*) and *N*_L_ = [N_L_](*t*). The evolution of the system 1–3 in time given the external signal *L*_air_ is fully described by five first order ordinary differential Equations 4–8 and two conservation Equations 9 and 10:
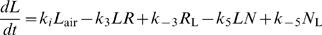
(4)

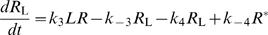
(5)

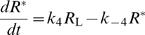
(6)

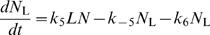
(7)

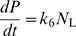
(8)

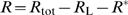
(9)

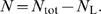
(10)Equations 9 and 10 follow from the fact that the total concentration of the receptor molecules, *R*_tot_ = *R*+*R*_L_+*R* ^*^, as well as the total concentration of the deactivating enzyme, *N*_tot_ = *N*+*N*_L_, do not change over time. We assume that at *t* = 0 the concentrations *L, R*_L_, *R* ^*^, *N*_L_ and *P* are zero. The parameter values, derived from extensive experimental investigations, are given in Table 1.

**Table 1:** Parameters of the perireceptor and receptor model.

Parameter	Value	Unit	Parameter	Value	Unit
*k*_3_ =	0.209	s^−1^µM^−1^	*k*_−3_ =	7.9	s^−1^
*k*_4_ =	16.8	s^−1^	*k*_−4_ =	98	s^−1^
*k*_5_ =	4	s^−1^µM^−1^	*k*_−5_ =	98.9	s^−1^
*k*_6_ =	29.7	s^−1^	*k_I_* =	29,000	s^−1^
*R*_tot_ =	1.64	µM	*N*_tot_ =	1	µM

From [25],[26].

### Basic Stimulus-Response Properties

The efficiency of information transfer in the system 1–3 depends critically on its stimulus-response relationship under single and repeated stimulus pulses. For transferring as much information as possible the response states must be optimally utilized. The actual amount of information transferred is limited by biological constraints. In the system studied, information transfer from *L*_air_ (stimulus) to *R* ^*^ (response) presents three main limitations.

First, it is limited by the finite number of receptor molecules per neuron which places an upper bound on the range of responses. Whatever the pheromone concentration (height of the step) the concentration of activated receptors cannot exceed 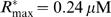 at any time [26].

Second, temporal details in the stimulus course shorter than a certain lower limit Δ*t* cannot be analyzed by the system. The smallest period of stimulation of the model studied here is 0.4 s [26],[27], in agreement with experimental measurements [28],[29]. With smaller periods, at higher frequencies, the amplitude of the oscillations of *R* ^*^ becomes too small to be effective. Therefore we set Δ*t* = 0.4 s. Two successive pheromone pulses separated by a time shorter than Δ*t* cannot be distinguished.

**Figure 3:**
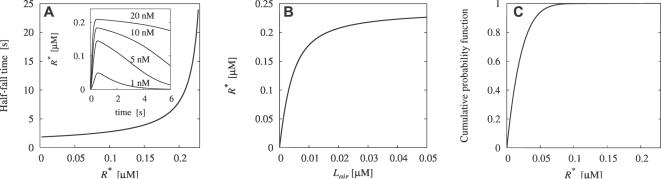
Response properties of the olfactory reception model. (A) Temporal properties. Inset: concentration of activated receptors, *R* ^*^(*t*), as a function of time for single pulses of pheromone of fixed duration (0.4 s) and different intensities *L*_air_ (1, 5, 10 and 20 nM). The maximum of *R* ^*^(*t*) is reached slightly after the end of the stimulation. The prolongation of the falling time with increasing intensities is quantified by the half-fall time, *τ*, as a function of *R* ^*^ at the end of stimulation. (B) Stimulus-response function *R* ^*^(*L*_air_) for single pulses of the same duration as in (A). This curve depends on the temporal resolution and the choice of the response intensity. (C) Optimal cumulative distribution function of the responses, *F_R_*(*R* ^*^), determined by maximizing the information transfer per average half-time (see Methods). The functions *R* ^*^(*L*_air_) and *F_R_*(*R* ^*^) were used for calculating the optimal stimulus probability distribution (shown in Figure 5B).

Third, information transfer in time is also limited by the response duration, which depends on the deactivation rate of the activated receptors. The time course of *R* ^*^ in response to stimulations of different heights *L*_air_ and limited duration (0.4 s) is shown in the inset of Figure 3A. The concentration of activated receptors rises at first, reaches *R*_Δ_^*^ at the end of the stimulus pulse, i.e., *R*_Δ_^*^ = *R* ^*^(*t* = Δ*t*), and finally decreases. We consider *R*_Δ_^*^ as the “response” of the system and for the sake of simplicity in the following, we omit index Δ. The duration of the falling phase (receptor deactivation) gets progressively longer for higher pheromone concentrations. This deactivation takes typically much longer than the time resolution parameter Δ*t*. The falling phase is often described by the half-fall time, *τ*(*R* ^*^), which is the time required for *R* ^*^(*t*) to decrease from *R* ^*^ to *R* ^*^/2. The relationship between *R* ^*^ and *τ*(*R* ^*^) is shown in Figure 3A. A unique value of *R* ^*^ corresponds to each value *L_air_*, which defines the stimulus-response curve (Figure 3B). The fact that the deactivation of activated receptors is relatively slow suggests that the reception system cannot encode a long sequence of pheromone pulses in arbitrarily quick succession. This observation plays an important role in the definition of the optimal stimulus course.

### Optimal Stimulus Course

In the simplest scenario (with no other constraints on the response range and stimulus-independent additive noise), the inputs should be encoded so that all responses are used with the same frequency [2],[30]. The optimal stimulus is thus described by its probability distribution function, which is obtained directly from the stimulus-response curve. Due to the large differences in reaction times at different stimulus concentrations, all response values *R* ^*^ from 0 to 0.24 µM cannot be considered as equally “usable” (the long falling phases decrease the efficacy of the information transfer). Therefore, the longer the half-fall time of a given response *R* ^*^ (i.e. the greater concentration *R* ^*^ is) the less frequent it must be. The particular form of the optimal response cumulative probability distribution function (CDF), *F_R_*(*R* ^*^), which was determined by maximizing the information transferred and minimizing the average half-fall time (see Methods), is shown in Figure 3C. Then, based on the three factors mentioned (stimulus-response curve, Figure 3B; time resolution Δ*t* = 0.4 s; and optimal response probability distribution, Figure 3C), an optimum stimulus course in time can be predicted as explained in the Methods section.

**Figure 4:**
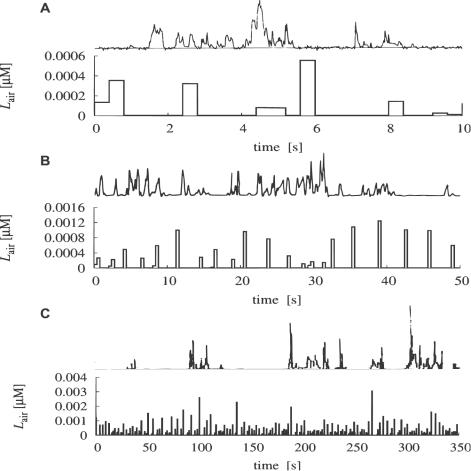
Qualitative comparison of reconstructed optimal pheromone stimulations *L*_air_ with experimentally-measured fluctuations in concentration of tracers (in arbitrary units), at various time scales. 10 s (A), 50 s (B) and 350 s (C). Temporal positions of pulses in experiments and simulations do not need to coincide. Quantitative comparisons are done in Figures 5 and 6 and in Table 2. (A) Ion signal measured using Langmuir probe in the field, 2.5 m from the source (top, from [17]); theoretical prediction (bottom) shows reasonable correspondence: the temporal resolution Δ*t* = 0.4 s is sufficient to capture the main bursts of pheromone. (B) Ion signal, averaged over 330 ms, distance up to 30 m from the source (top, from [39]); the predicted signal (bottom) captures the overall character of the natural stimulation. (C) Propylene source, 67 m from the source (top, from [18]); the longest pauses (over 1 minute) are caused by the global meandering of the plume: they are absent in the prediction (bottom) because moths are assumed to stay within the pheromone plume.

Examples of predicted temporal fluctuations in pheromone concentration are shown in Figure 4 at various time scales and compared to experimental observations. Even though the time resolution of the system studied here is only 0.4 s, it seems sufficient to capture the main bursts of pheromone (see the 10 s sample in Figure 4A). The comparison can be made more precise by describing statistically the heights and occurences in time of the pulses.

**Table 2:** Comparison of statistical characteristics of optimal and actual plumes.

Characteristics^a^	Predicted Values^b^	Experimental Values
Concentration CDF (Figure 5)	Exponential	Exponential [18],[19],[32]
Spectra (Figure 5)	Approx. flat to 0.2 Hz,	Approx. flat to 0.1 Hz or 0.5 Hz
	Close to −2/3 slope after	−2/3 slope to 1 Hz [19],[33])
Intermittency	20%	10–40% [17],[39]
		10–20% [15]
Total mean *L*_air_	1.0×10^−4^ µM	–
Total std. dev. of *L*_air_	3.0×10^−4^ µM	–
Peak value of *L*_air_	3.8×10^−3^ µM	–
Peak/mean ratio	37	>20 [17],[39]
		30–150 [15]
Peak/std.dev. ratio	13	>3 [18]

a The mean concentration, standard deviation and their ratios are calculated from the complete stimulus course, including parts of zero concentration (see Methods).

b Based on a simulated sample 4000 s long.

### Predicted Temporal Pattern of Pulses

Concerning temporal aspects, the bursts of non-zero signal do not occur at periodic intervals but appear randomly. An important descriptor of the temporal structure is the intermittency [15],[16], which is the fraction of total time when the signal is present. The intermittency of the predicted optimal stimulus is 20%, which is in relatively good agreement with experimental data. It has been shown using various types of ion detectors [17],[19] as well as electroantennogram responses [17],[31], that the natural signal is always present less than 50% of the total time, and usually smaller values are found. The average intermittency values reported are 10–20% [15] and 10–40% [16],[17], depending on the experimental conditions, such as the detector size or the global meandering of the plume (see Discussion).

### Predicted Concentrations of Pheromone Pulses

Concerning pulse height, the overall character of the predicted stimulus course is that pulses of high concentration are much rarer than those of low concentration. This feature of the predicted stimulus can be best quantified by the CDF, *P*(*L*_air_), of the stimulus. The shape of the CDF is one of the most important properties for comparing theoretical predictions to experimental measurements because it describes the relative distribution of odorant concentrations throughout the plume. In fact, because measuring pheromone concentration in the field is not presently feasible [17], pheromone molecules must be replaced by measurable tracers. Relative quantities are valid for both pheromones and tracers (see Discussion). They are the only quantities known experimentally for pheromone plumes. So, although our model predicts them, we cannot compare values of *L*_air_ to actual measurements.

**Figure 5 pcbi-1000053-g005:**
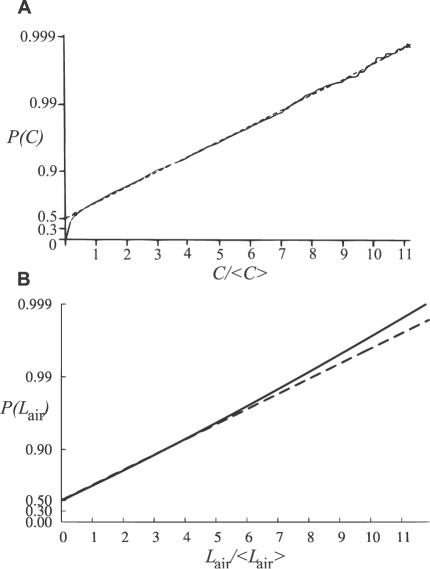
Experimental and theoretical cumulative probability distribution functions (CDF) *P*(*C*) of dimensionless odorant concentration *C/*<*C*> (concentration divided by the total mean concentration). (A) Experimental CDF (solid) as measured at 75 m from a propylene (passive tracer) source and its best exponential fit (dashed) plotted on a logarithmic scale (taken from [19]). The intermittency is included in the plots in the non-zero value of *P*(*C*) for zero concentrations (see Methods). The experimental data clearly follow the exponential CDF, except close to *C* = 0, which is caused by technical issues in the measurement process [19]. The relatively high value of measured intermittency (close to 47%) is caused mainly by initial data processing [19]. (B) CDF predicted by the pheromone reception model together with its best exponential fit, the scales correspond to panel (A) After correcting (see Methods) for the fact that the intermittency predicted by the pheromone reception model (20%) is lower than that measured in [19] (as explained in the Discussion), the predictions correspond well to the measured data in (A), except at very high values of *L*_air_ where they are less frequent than expected. Since this deviation is apparent only for events occurring with probability *P*<0.01, it can be considered as non-significant.

Given the definition of the optimal stimulus, function *P*(*L*_air_) can be directly computed (see Methods). Figure 5 shows a comparison between experimentally measured (A) and predicted (B) concentration CDF. The optimal pheromone concentration CDF (Figure 5B, solid line) is not known in analytical form but it can be well approximated by an exponential CDF (Figure 5C, dashed line). The differences between the predicted and true exponential shape can be considered as non-significant, namely, very high values of *L*_air_ are predicted to be less frequent than in the exponential model. The exponential CDF is in agreement with experimental CDF (Figure 5A), [18],[19],[32],[33] and holds well especially for observations closer to the source (less than 100 m). Although the precise form of the CDF varies with distance from the plume centerline [19] and may be affected by the measurement technique, the shape is always highly skewed.

Other predicted relative quantities (peak-to-mean ratios, dimensionless concentrations *L*_air_/〈*L*_air_〉) were compared with their experimental counterparts. The results, summarized in Table 2, show that the predicted statistical properties of the stimulus are not contradicted by the experimental observations.

### Spectral Density Functions of the Stimulus Course

Spectral density functions of the concentration time course, which analyze the contribution of various frequencies to the overall stimulus course, characterize other properties of the plume which are independent on the nature of the odorant (pheromone or ion source) [19],[33]. Furthermore, spectral density function represents a point of view different from the concentration probability distribution.

**Figure 6:**
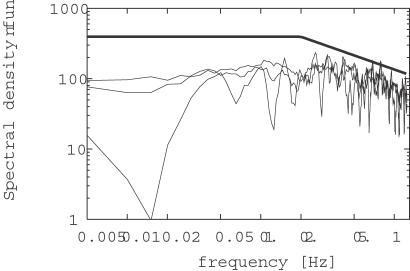
Spectral density functions of the predicted fluctuations in time of pheromone concentration. Several spectral density functions were calculated from the predicted optimal pheromone stimulations (such as shown in Figure 4, bottom panels), for different initial random seeds. Calculated spectral shapes are usually almost flat from 0.02 Hz to 0.2 Hz, although exceptions are sometimes observed at lower frequencies, which are also found in experimental data [19]. Above 0.2 Hz there is a decreasing slope close to −2/3. Flat spectrum up to 0.2 Hz and true −2/3 slope beyond are shown for comparison (thick line). Spectra from experimental measurements (not shown) on propylene plume obtained close to the source are reported to exhibit a similar flat region followed by −2/3 slope [19].

Several spectral density functions, shown in Figure 6, were calculated from the predicted optimal pheromone stimulation (see Methods). The spectral shapes seem to be almost flat from 0.02 Hz to 0.2 Hz with a decreasing slope close to −2/3 above 0.2 Hz. The same slope −2/3, which is theoretically predicted by the inertial subrange theory [19], was reported in the spectral densities obtained from measurements close to the source (less than 100 m), in the range 0.1 Hz (or 0.5 Hz, depending on records) to 1 Hz [19],[33], although the precise range may depend on the technique of measurement.

## Discussion

The goals of this study were to determine to which extent early olfactory transduction in olfactory receptor neurons can be considered adapted (in the evolutionary sense) to odorant plumes and to specify the plume characteristics to which it is adapted. The formulation and resolution of this problem benefited from successful studies of efficient sensory coding undertaken in the field of vision and audition. However, transposition from these sensory modalities to olfaction is not straigthforward, which may explain in part why it has not been attempted earlier. Specificities of olfaction concern both the odorant plume and the sensory system.

### Odor Plumes

In theory and in practice, the quantitative description of odor plumes and their spatiotemporal distribution is less straightforward than that of visual or auditory scenes. Contrary to light and sound, for which the physical description is essentially complete, the turbulent phenomena which underlie the plume characteristics are still an incompletely mastered domain of physics [34].

In Laughlin's classical experiment in vision a single time-independent variable, the contrast level, was measured [2] and directly compared with experimental data. In olfaction, however, the odorant concentration (an analogue to the contrast level) is essentially time dependent which results in a complex optimal stimulus course (Figure 4). Complexity and time dependence make a meaningful direct comparison between predictions and experimental records, but also between different experimental records, impossible. Instead, the comparison must rely on global, statistical descriptors [15],[17],[19],[33]. We identified 5 such descriptors of odor plumes, actually measured and usable in the present context (see Table 2), which summarize the present knowledge on odor plumes.

Moreover, there are no easy-to-use instruments to measure odor plumes in the field, comparable to luxmeters and microphones. For example, the absolute pheromone concentration cannot be easily known in field experiments [17]. This explains why no experimental values were given for this descriptor in Table 2. In practice, only ratios of concentrations are presented because they are independent of the dispersed molecules. The pheromone is often substituted by an ion or a passive tracer (polypropylene for example) whose concentration can be measured [15],[17],[19]. Because both pheromone and tracer compounds in the air are governed by the same physical laws, the relative (dimensionless) values are conserved, as confirmed by independent experiments with different sources [15]–[17],[33]. More generally, this limitation explains why we compared only relative quantities (i.e. shape of probability distributions, spectral density functions, peak-to-mean ratios, dimensionless concentrations *L*_air_/〈*L*_air_〉 and intermittency values). Other limitations of plume measurements are discussed below.

### Model of Early Transduction

The essentially multidimensional and stochastic nature of the odor stimulus has a profound influence on the analysis of olfactory transduction system in its natural context, as undertaken here. Indeed to investigate the problems at hand, the kinetic responses of the system to a very large number of stimuli, varying in intensity, duration and temporal sequence must be known in order to simulate the diversity of stimuli encountered in a natural plume. This task is difficult, if not impossible, to manage in a purely experimental approach. However, this difficulty can be overcome with an exact dynamic model of the system because its response to the diverse conditions mentioned can be computed, provided it includes all initial steps from molecules in the air to the early neural response. This is the case of the perireception and reception stages of the moth pheromonal ORN and the reason why it was chosen in the present study. This choice brings about two questions, one about the validity of the model, the other on its position within a larger context.

The computational model employed has been thoroughly researched and improved over the last three decades [25], [35]–[37]. It describes perireceptor and receptor events in the ORN cell type sensitive to the main pheromone component of the saturniid moth *Antheraea polyphemus*. At the time of writing it represents the most completely researched computational models of its kind, agreeing with extensive experimental data from various authors and a wide range of experimental techniques. This model is the best description presently available for early events in any ORN and it summarizes in a nutshell a wealth of dispersed knowledge. This model is based on ordinary differential equations 4–8, following the law of mass action for chemical reactions, and is therefore purely deterministic. This approximation is acceptable when the concentrations of reactants are high enough above single-molecular levels, so that the stochastic fluctuations can be neglected. In this paper, the concentration of *R* ^*^ is always well above that corresponding to one activated receptor molecule per neuron (approximately 10^−6.2^ µM) because we do not investigate the effect of extremely small pheromone doses. Then, the response of the system can be considered as deterministic, in accordance with the efficient coding hypothesis [8].

The system studied here constitutes only a small part of the whole pheromonal system, although its role is absolutely essential and all other parts depend on it. First, in ORNs, post-receptor mechanisms modify the receptor signal, primarily by a large amplification factor and by sensory adaptation. Second, the ORN population includes cell types with different properties, e.g. the ORN type sensitive to the minor pheromone components can follow periodic pulses up to 10 Hz [29], a performance not yet accounted for in present models [27]. Third, in the brain antennal lobe, convergence of a large number of ORNs on a few projection neurons (PNs) provides another amplification and supports the ability of some PNs to follow periodic signals at 10 Hz or greater [38]. Evolutionary adaptation of an integrated ORN response is difficult to study at the present time because no complete model of the ORN from receptors to the generation of the receptor potential and the ensuing spike train, is yet available, at least with the required degree of precision. The same argument holds a fortiori for higher order processes. Notwithstanding, the study of the early sensory events is not as restrictive as it may seem because any incoming odor signal must be first transduced in the population of membrane receptors. No information can be extracted by the post-receptor transduction system which has not been encoded by the receptors in the first place. For this reason it is essential to investigate the nature of the adaptation of the initial events (pheromone interaction with receptors) to the pheromone signal.

### Determination of the Optimal Stimulus

Different response states of the pheromone reception system have different efficacies from the coding point of view: the “high” states, with large concentrations of activated receptors, take much more time to deactivate than the “low” states, so that for some time after its exposition to a large concentration of pheromone the system is “dazzled”. It means that in the optimal stimulus the low pheromone concentrations must be more frequent than the high ones. This is a difference with respect to the classical problem where the efficacy of all response states at transferring information is considered the same, as in the vision of contrasts for example. The problem to solve is to find the right balance between two conflicting demands: to use all response states (including the high ones) and to react rapidly (the short transient responses must be as frequent as possible), i.e. to maximimize the information transferred *per time unit*.

The solution to this optimization problem is provided by information theory as detailed in the Methods section. The optimal balance derives from Equation 19 which relates the average half-fall time and the maximum response entropy distribution. The key factor to consider in the optimization is the average half-fall time, which characterizes globally the “swiftness” of the system – smaller average half-fall time means faster stimulation rate. In other words, the average half-fall time characterizes the bias towards “low” response states. Simultaneously, the condition of maximum response entropy guarantees that the temporal dynamics of the system is as varied as possible and that during the course of stimulation every possible response state is used (with appropriate frequency). By taking into account only the average half-fall time, and not the precise sequence of its individual values, we therefore do not neglect or limit the temporal dynamics of receptor molecules activation. It is important to note, that the average half-fall time is not a free parameter of the problem; it is not set a priori: its optimal value follows from the optimization procedure (Equation 20). The resulting optimal response CDF is highly biased towards low response states, as expected (see Figure 3C).

### Nature of the System Adaptation

The main achievement of the present investigation was to predict the characteristics of the stimulus optimally processed by the receptor system based on its biochemical characteristics and an information theoretic approach. The predicted optimal plume was shown to be close to the actual plumes for a series of characteristics, namely intermittency, peak/mean ratio and peak/standard deviation ratio of pheromone pulses, probability distribution of dimensionless pheromone concentration and spectral density function of pheromone concentration (Table 2, Figures 4–6). The correspondence between the predictions and measurements is very good for the last two characteristics (probability distributions) and fair for the first three (numerical values).

These differences in precision of the predictions may be interpreted by taking into account technical factors. Increasing the noise rejection threshold leads to a decrease of the measured intermittency [15],[19], while increasing the detector size or averaging the signal over longer time windows has the opposite effect [39]. So, for example, the small size of olfactory sensilla with respect to detectors may explain in part why in Figure 4B, the predicted intermittency seems lower than that in the corresponding experimental record sample, and also why the peak-to-mean ratio and peak-to-standard deviation ratio are relatively higher. The immobility of the measurement devices, in contrast with the active movements of the moths, is another significant factor. For example, long pauses (of the order of minutes) of zero signal are missing in the prediction but visible in the longest available field record (350 s, Figure 4C). They are caused simply by the plume being blown away from the immobile field detector. First, this loss of signal is clearly an extraneous effect, which cannot be included in our optimal signal predictions and therefore cannot be seen in our results. Second, the moth is not subjected to this extraneous effect, or at least not to the same extent, because, in case of signal loss, it actively seeks the pheromone plume, whereas the fixed detector must passively wait for its return. This difference of mobility may substantially affect the intermittency values, but does not affect the shape of probability distributions (see Methods), hence the better quality of the fits in the latter case. In conclusion, the results obtained suggest that the perireceptor and receptor system investigated here is evolutionary adapted to the pheromone plumes.

Even if one considers that the pheromone olfactory system must be *a priori* adapted to the average characteristics of the pheromone plumes, it does not logically follow that the system studied is itself necessarily well adapted. Indeed, it is conceivable that the global adaptation results mainly, not from perireception and reception processes but from other downhill intra- and intercellular processes involved in higher signal processing. The respective importance of the former and latter processes in global adaptation cannot be decided *a priori*. Therefore, the relatively close correspondence between predicted and observed plume characteristics presented here is not trivial. It suggests that the adaptation at the level of receptors is already substantial, and consequently that the global adaptation is not predominantly the result of post-receptor mechanisms involving amplification, sensory adaptation, convergence of different ORN types in the antennal lobes etc. The role of these mechanisms in the global adaptation of the animal remains to be established, as well as the relative importance of the various components of the olfactory system (receptor population, ORN as a whole, population of pheromonal ORNs in the antenna, projection neurons in the antennal lobes, etc.). The response characteristics of these other subsystems, e.g. their various temporal resolutions, will have also to be interpreted, maybe in relation with changing plume characteristics with distance to the source and other factors yet to be identified.

## Methods

### Optimal Response Probability Distribution Function

As mentioned in the Results section, information transfer in the pheromone reception system is limited by the finite response range, (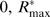), and by the deactivation rate of the activated receptors for each concentration value *R* ^*^. This deactivation rate is described by the half-fall time *τ*(*R* ^*^). The optimal performance of the system is thus reached by a trade-off between two conflicting demands: to employ full response range (maximum information) vs. to employ only the “fastest” responses (minimum average half-fall time). In other words we need to maximize the information transferred per average half-fall time. In the following we provide the mathematical framework that enabled us to find the probability distribution function over the response states *R* ^*^ that realizes this trade-off.

#### Information transferred

The information transferred by the pheromone reception system in a selected time window (*t,t*+Δ*t*) is described by the relation between all possible stimulus values, *L*_air_, and the corresponding response values, *R* ^*^. This relation is explicitly quantified by the mutual information, *I*(*L*_air_; *R* ^*^) (see [13] for details)
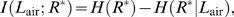
(11)where *H*(*R* ^*^) is the entropy of the response probability distribution function and the conditional entropy *H*(*R* ^*^|*L*_air_) measures the uncertainty in the output given the input, or equivalently, the amount of noise in the information transduction [13],[14]. The model of pheromone reception employed here is deterministic and therefore *H*(*R* ^*^|*L*_air_) = 0. Thus maximizing the mutual information corresponds to maximizing the response entropy *H*(*R* ^*^). (Note that in the usual setting of signal independent and additive noise the term *H*(*R* ^*^|*L*_air_) is constant and then maximization of *I*(*L*_air_;*R* ^*^) again corresponds to maximization of *H*(*R* ^*^).)

The available response range, (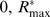), is naturally discrete, since it is comprised of individual receptor molecules. The expression of *H*(*R* ^*^) is ([13], p.14)
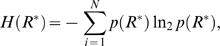
(12)where *p*(*R* ^*^) is the probability of having *R* ^*^ (expressed as a number of molecules). (In the following we use the base of logarithm 2 only to express all information-related quantities in the usual units of “bit”).

The value of *R* ^*^ corresponding to one activated receptor molecule per neuron is approximately Δ*R* ^*^ = 10^−6.2^ µM [26], which gives a total of 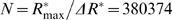 different response states. Since *N* is so large, the impractical Equation 12 can be replaced by a continuous approximation based on differential entropy, *h*(*R* ^*^), defined as ([13], p.243)
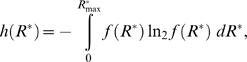
(13)where *f*(*R* ^*^) is the response probability density function. An approximative relation between *H*(*R* ^*^) and *h*(*R* ^*^) is given in ([13] p.248)
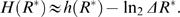
(14)In the present case the approximation is excellent because the discretization step Δ*R* ^*^ is very small compared to the whole response range (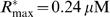). From relation 14 the mutual information 11 can be expressed in terms of differential entropy
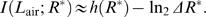
(15)Maximizing the information transferred is thus achieved by maximizing the differential entropy *h*(*R* ^*^). The advantage of employing differential entropy is that it lends itself to an elegant approach for entropy maximization in terms of integrals.

#### Information optimization

We adopt the standard procedure for maximizing the differential entropy of a continuous probability distribution constrained by a known function *τ*(*R* ^*^). “Constraining” means that the average value 〈*τ*〉 of *τ*(*R* ^*^) is under our control (see [13], p.409)
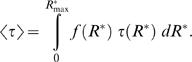
(16)The task is to find a probability density function, *f_R_*(*R* ^*^), which (i) maximizes the value of *h*(*R* ^*^) (Equation 13) and (ii) is such that the average 〈*τ*〉 (Equation 19) taken over *f_R_*(*R* ^*^) is equal to the value we set. The well known solution to this problem (see [13], p.410 or [40] for its derivation) is
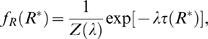
(17)where
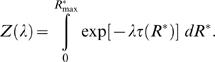
(18)It depends on new parameter, *λ*, called Lagrange multiplier. In the standard setting of maximum-entropy problems ([13] p.409 or [8],[40]) the mean value of the constraint function, 〈*τ*〉, is known *a priori*. The value of *λ* is then determined by substituting *f*(*R* ^*^) = *f*_R_(*R* ^*^) in Equation 16, so that the following equation between 〈*τ*〉 and *λ* holds
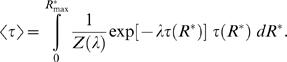
(19)In the case of pheromone reception, however, the value of 〈*τ*〉 and consequently of *λ* is unknown. The value of *λ* must be determined by finding a compromise between maximum information transferred (Equation 15) and minimum average half-fall time (Equation 19). This compromise is made explicit by a simple requirement
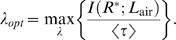
(20)In other words we maximize the information transfer per half-fall time.

#### Application to pheromone reception

**Figure 7:**
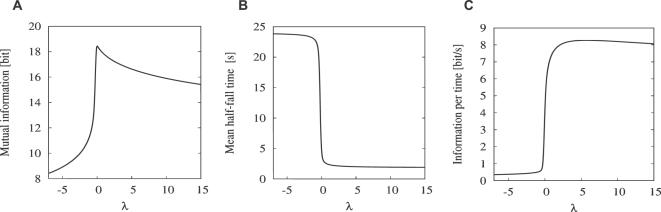
Determination of the optimal Lagrange multiplier *λ* giving the optimal response probability distribution. (A) Mutual information for the pheromone reception model in dependence on *λ* (Equation 21). (B) Mean half-fall time in dependence on *λ* (Equation 19). (C) Information transferred per average half-time representing the balance between the reactivity of the system and information transferred (Equation 20). Maximum occurs for *λ*≈6.

In order to simplify practical calculations we substitute *f*(*R* ^*^) = *f_R_*(*R* ^*^) into the definition of differential entropy 13 so that Equation 15 reduces to
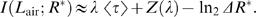
(21)Now we have all the necessary information to calculate (i) the mutual information *I*(*L*_air_;*R* ^*^) from Equation 21 (shown in Figure 7A), (ii) the mean half-fall time from Equation 19 (Figure 7B) and (iii) their ratio from Equation 20 (Figure 7C) in dependence on the Lagrange multiplier *λ*. Figure 7A shows that the mutual information is maximized (18.5 bits) for *λ* = 0 which corresponds to the uniform probability distribution function over the whole response range. Generally, since *τ*(*R* ^*^) is a monotonously increasing function of *R* ^*^, the optimal probability density function *f_R_*(*R* ^*^) (Equation 17) is either monotonously increasing (*λ*<0), monotonously decreasing (*λ*>0), or constant (*λ* = 0). The multiplier *λ* thus decides whether *f_R_*(*R* ^*^) puts more weight on the “slow” response states (*λ*<0) or on the “fast” response states (*λ*>0). These observations are confirmed in Figure 7B where the mean half-time monotonically decreases with increasing *λ*. Figure 7C shows the information transferred per average half-time, i.e., it shows the compromise between the “slowness” or “reactivity” of the system and the transferred information. Clearly, there cannot be a maximum for *λ*<0 where the system is both “slow” and below its information capacity (note the sharp decrease of mutual information in Figure 7A for *λ*<0). The optimal balance between reactivity and information transfer is reached for *λ*≈6 at 8 bits/s. By substituting *λ* = 6 into formula 17 we obtain the desired optimal response probability density function, *f_R_*(*R* ^*^), which maximizes the information transfer per average half-time. The corresponding CDF *F_R_*(*R* ^*^), shown in Figure 3C, is given by
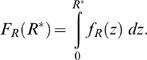
(22)


The maximum of information transferred per average half-time (Figure 7C) is not sharply defined, namely, the transfer of 7–8 bits/s persists with values of *λ* greater than the optimal value. At the same time, both mutual information (Figure 7A) and average half-time (Figure 7B) decrease slowly in the corresponding region, indicating that the shape of the optimal response probability distribution changes slowly with respect to *λ*. Indeed, as we verified numerically, varying *λ* within reasonable limits (so that information transferred stays close to 8 bits/s) has no impact on the results presented in this work.

### Optimal Stimulus Course

The optimal stimulus course in time was calculated as follows. First, at time *t*_0_ = 0 a random value *p*_0_ is drawn randomly from a uniform probability distribution function over the range [0,1]. The concentration 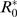 corresponding to probability *p*_0_ is obtained by solving the equation
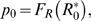
(23)where *F_R_*(*R* ^*^) is the optimal CDF given by formula 22 (Figure 3C). The predicted optimal concentration *L*_air,0_ for a pheromone pulse of duration Δ*t* = 0.4 s which corresponds to 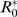 is obtained by solving the equation
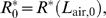
(24)where *R* ^*^(*L*_air_) is the stimulus-response function (Figure 3B). The value *L*_air,0_ is plotted at *t*_0_ (Figure 4). Second, the concentration *L*_air,1_ and time of appearance *t*_1_ of the next pulse are determined. Time *t*_1_ follows from the falling phase of activated receptors: optimality requires that no pheromone pulse appears before *R* ^*^ returns to its resting level. In practice it is considered that the resting level is reached when *R* ^*^ falls below 0.01 µM (less than 5% of the coding range). The concentration *L*_air,1_ of the pulse at *t*_1_ is determined in the same way as for the pulse at *t*_0_ by drawing a new random number *p*_1_ from the uniform probability distribution function over [0,1]. The same process can be repeated as many times as needed to create an optimal pheromone pulse train of arbitrary length.

### Optimal Stimulus Probability Distribution Function

It is common in the literature on the statistical analysis of plumes [15],[18],[19] to define two types of mean concentrations. The total mean concentration, 〈*L*_air_〉, describes the “true” mean concentration obtained from the whole record of concentration fluctuations in time, i.e., including the parts where no signal was available. On the other hand, the conditional mean concentration, 〈*L*_air_〉_cond_, describes the mean concentration inside the plume, i.e., with zero concentrations excluded. The intermittency, *γ*, relates the two means as [19]

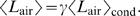
(25)(Analogously, the total variances and total standard deviations are calculated by taking into account also the parts where no signal is available [19].)

By combining Equations 23 and 24 we may symbolically express the optimal CDF of the stimulus, *P*(*L*_air_), as
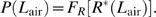
(26)Though *P*(*L*_air_) cannot be expressed in a closed form, it can be well approximated by the exponential CDF
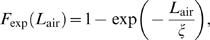
(27)where *ξ* = (5.24±0.01)×10^−4^ µM is the estimated value of 〈*L*_air_〉_cond_ by least-squares fitting of *F*_exp_(*L*_air_) to *P*(*L*_air_).

In order to compare concentration probability distribution functions from different measurements meaningfully, authors [19] plot the CDF for a dimensionless concentration *L*_air_/〈*L*_air_〉. (In the Figure 5A *C*/〈*C*〉 is used, since the data plotted were obtained using a propylene source, not pheromone), see Figure 5A. The scale of such plots is affected by intermittency due to the presence of the total mean in the ratio. Furthermore, information about intermittency is included explicitly in the plots by letting the probability *P*(*L*_air_ = 0) of zero concentration be
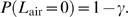
(28)Consequently the CDF *P*(*L*_air_) must be renormalized [19]. Intermittency affects only the dimensionless scale, *L*_air_/〈*L*_air_〉, and the value of *P*(*L*_air_ = 0) but not the overall shape of CDF [19]. Therefore we can use formulas 25 and 28 to compare our predictions with experimentally measured data by correcting for different intermittency values.

### Spectral Density Function of the Stimulus Course

The optimal stimulus course is represented by pulses of different pheromone concentrations, *L*_air_, occurring in time intervals 0.4 s long. In order to calculate the spectral density function of such stimulation course we sample the time axis with step Δ*t* = 0.4 s. Thus we obtain a series of pheromone concentrations at these time points, {*L*_air,*j*_}, *j* = 1*…n*, where *n* should be even. The discrete Fourier transform, *φ_k_*, of {*L*_air,*j*_} is defined for *k* = 1,*…,n* values as [41]

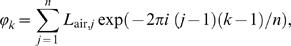
(29)where *i* is the complex unit. The zero-frequency term is thus at position *k* = 1. The spectral density, *S*(*f*), of the complete time course of the stimulus can be calculated for a total of *n*/2+1 values of frequency *f* (given in Hz) [42]

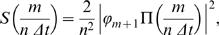
(30)where *m* = 0, 1, 2,*…, n*/2−1, *n*/2 and *f* = *m*/(*n*Δ*t*) are the frequency values. The function Π(*f*) is the Fourier transform of a pulse of unit height, 0.4 s long and starting at *t* = 0 [41],
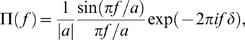
(31)where *a* = 2.5 and *δ* = −0.5. The function Π(*f*) appears in formula 30 because the whole stimulus course (such as shown in Figure 4, bottom panels) can be reconstructed by convolving the discrete series {*L*_air,*j*_} with such a pulse of unit height in the time domain [41].
